# “It started 30 years ago, and it still haunts me”: an exploratory investigation of Territorians’ gambling behaviours, harm, and help-seeking for gambling issues in an Australian jurisdiction

**DOI:** 10.1186/s12889-020-10141-5

**Published:** 2021-01-07

**Authors:** Himanshu Gupta, Matthew Stevens

**Affiliations:** 1 Menzies School of Health Research, Charles Darwin University, PO Box 41096, Casuarina, NT 0811 Australia; 2 National Drug Research Institute, Curtin University, GPO Box U1987, Perth, WA 6845 Australia

**Keywords:** Gambling, Northern Territory, Australia, Lived experience, Regulation

## Abstract

**Background:**

There is a lack of qualitative gambling research on lived experience, help-seeking, and gamblers and affected others’ views on the regulatory environment in the Northern Territory (NT), Australia. This study provides 1) lived experience of individuals who reported experiencing harms from gambling, 2) insights into help-seeking for gambling issues, 3) and people’s views on current legislation on gambling in the NT. The results of this study begin to establish an evidence base that could be used to inform targeted interventions for people experiencing harms from gambling in the NT.

**Methods:**

Semi-structured interviews were conducted with a targeted selection of respondents from the 2015 and 2018 NT Gambling Prevalence and Wellbeing Surveys. The sample (*n* = 27; age 18+ years; Aboriginal (The term of ‘Aboriginal’ has used been used throughout the manuscript to reflect Aboriginal and Torres Strait Islander, Indigenous, or First Nations people for purposes of brevity. We respect the diversity among these populations.) and Non-Aboriginal) included weekly (online and venue-based electronic gambling machine (EGM)) gamblers, non-regular gamblers, and those negatively affected by others’ gambling. A Framework Analysis approach was used for data analysis. Appropriate ethics approval was obtained.

**Results:**

Negative impacts and harms from gambling were experienced by both gamblers and non-gamblers. These included monetary losses, relationship conflicts, emotional distress, and decrements to health. A lack of self-realisation of gambling issues and awareness of the available services, shame, and embarrassment, were reported as the main barriers to help-seeking. Where help was sought, it was primarily informal (e.g., family) and was rarely preventive. In many instances, self-help strategies were successful in controlling one’s own gambling. Gamblers suggested regulations should set limits on the daily number of hours of playing, the bet size, and reduced access to EGM. The need for strengthening the existing awareness and education interventions was emphasised.

**Conclusions:**

Viewing the findings from a public health lens, targeted approaches based on specific circumstances may have the potential to minimise harms from gambling, but only for those already experiencing harms. The treatment, policy, and regulatory approaches need to be tailored to address the causes and impacts of harms experienced by people.

**Supplementary Information:**

The online version contains supplementary material available at 10.1186/s12889-020-10141-5.

## Background

Australia has one of the highest gambling participation rates in the world [1]. An estimated 64% of the adult Australian population take part in some form of gambling annually [2], of whom about 2.5% of experience moderate to severe problems caused by gambling [1]. Further, for every problem gambler, about six others (such as family and friends) are negatively affected by their gambling activities [3]. This indicates that up to four million Australians experience harms from gambling including economic harm, decrements to physical and mental health, and adverse social impacts [4].

Given the unprecedented growth of the gambling industry and the high rates of gambling harm in Australia [1], the role of governments as a regulator has grown and the approaches to minimise harms from gambling have become more complex. Some of the recent gambling-related qualitative studies conducted in Australia (other than the Northern Territory (NT)) provide insights into people’s experiences with gambling-related harms and help-seeking, with a focus on exploring the potential of a targeted intervention approaches to minimise harms from gambling [5–7].

### Harms from gambling

In light of the Langham’s conceptual framework of gambling-related harms [4, 8], earlier studies have described harms from gambling in terms of financial harm, emotional distress and relationship conflicts, and decrements to health. Financial difficulties and negative emotional impacts (such as distress, guilt, shame, anger, and hopelessness), were identified as primary negative impacts of gambling [5–7]. Less common impacts were health impacts (such as a loss of sleep, eating problems, and increased alcohol consumption), relationship impacts (such as spending less time with family and friends, and tension in relationships), and impact on work/study [5–7]. Regardless of the direction of the relationship, the circular associations between gambling and these harms were identified.

### Help-seeking for gambling issues

Previous studies have identified that help-seeking was not commensurate with the rates of gambling-related harms and is rarely preventive. In many cases, gamblers were unwilling to seek help because of a lack of acceptance of the problem. Where substantial harms were experienced, help-seeking was still low, largely due of the shame and guilt associated with problem gambling and a lack of awareness about the professional services. Where help was sought, it was primarily through close personal contacts, such as family and friends. Professional sources of help included gamblers help line, Internet-based gambling help line, counsellors, and General Practitioners [5, 7].

### Self-help strategies

Previous research in Australia has found that many gamblers use self-help strategies to reduce their gambling issues. The strategies included creating barriers to access and spend money on gambling such as carrying set amount cash to venues, not carrying credits cards to venues, self-exclusion from venues, having partners take control of the finances, setting up direct debits which paid bills before the gambler had access to that money [5, 7]. Other strategies involved finding ways to consciously distract mind from gambling, such as getting involved with the local sports club for social interaction and escape boredom and loneliness [7].

### Gambling research in the NT

Most of the gambling-related research conducted in the NT appears to be quantitative [9–12]. There exist a few qualitative studies that have been conducted in the NT; however, they mainly focused on exploring gambling activities (less about commercially available gambling and more about cards) and related harms (especially, around problem gambling) in a few remote Aboriginal communities in the NT [13, 14]..

The most recent published report using data from the 2015 NT Gambling Prevalence and Wellbeing Survey found higher participation rates in keno, casino table games, sports betting, and any gambling in the NT compared with other jurisdictions in Australia [9]. The motivations for gambling were socialisation, money, and escape from problems. About 4% (*N* = 6500) of respondents were identified as experiencing moderate risk of or problem gambling (Problem Gambling Severity Index (PGSI) 3+). An estimated 13% (*n* = 23,000 people) of respondents indicated that they had experienced at least one negative consequence from someone else’ gambling in the past year [9]. The negative consequences were primarily financial (such as running out of money and borrowing money from family/friends), poor mental health (such as stress, anxiety, and depression), relationship impacts (such as tension in relationships) [9].

As stated above, compared with other Australian jurisdictions, NT has higher rates of gambling-related harm but low levels of help-seeking. For example, less than 5% of 9341 at-risk gamblers indicated that they sought help because of their gambling in the 2015 NT Gambling Prevalence and Wellbeing Survey [9]. The excessive prevalence of gambling problems in the NT is because of size of the gambling industry that caters for just about 1% of Australia’s population who lives in the NT. For such a small population, the NT has a gambling industry that accounts for more than 6% of Australia’s total regulated gambling turnover and more than 5% of total gambling expenditure [9]. Furthermore, the NT has a younger population than other Australian jurisdictions, and specific socio-demographics that are risk factors for gambling harm. These include a large Aboriginal population, many of which are socio-economically disadvantaged, a history of gambling in this population group, and also a large population of Fly-in Fly-out workers that have both significant incomes and time on their hands due to the nature of the work [15, 16]. Capturing lived experience of individuals experiencing harms from gambling, their opinions on and experience with help-seeking for gambling issues, and views on potential approaches to minimise harm form gambling, would, therefore, be useful for developing interventions and targeted treatment approaches to regulate gambling harm in the NT.

To address the paucity of qualitative gambling research in the NT, an exploratory qualitative study was conducted to 1) provide lived experience of individuals who reported experiencing harms from gambling, 2) insights into help-seeking for gambling issues, and 3) and people’s views on current legislation on gambling in the NT. The sample consisted of both gamblers and those who were negatively affected by someone else’s gambling (or affected others). The latter cohort of participants was included in the sample because 1) the 2015 NT Gambling Prevalence and Wellbeing Survey indicated that a substantial proportion (about 13%) of respondents reported that they had experienced at least one negative consequence from someone else’ gambling in the past year [9] and 2) most of the previous gambling research has primarily focused on gamblers and thus examining affected others experiences is vital when developing relevant interventions and targeted treatment approaches. It is anticipated that the results of this study will provide an evidence-base that can be used to develop targeted interventions and improve gambling policy to minimise gambling-related harms in the NT.

### Study objectives

- Capture lived experience of individuals^1^ experiencing harms from gambling.
- Provide insights into help-seeking for gambling issues, preferences for types of interventions, and the preferred contexts for offers of help.
- Explore the range of self-help strategies people use to control their gambling.
- Explore opinions on gambling policy and legislation and potential approaches to minimise harm from gambling in the NT.

## Method

### Sample selection

We used a purposive sampling method to recruit the participants. It enabled us to capture experiences of a variety of voices including a diverse representation of demographic characteristics. Participants were selected from the 2015 and 2018 NT Gambling Prevalence and Wellbeing Surveys (over 80% of participants in both surveys agreed to be re-contacted to participate in future research). An initial list comprised gamblers and people affected by someone else’ gambling was generated (*n* = 241). The gambling group consisted of both regular (weekly) gamblers and non-regular gamblers, and was split between EGM, sports, and racetrack gambling activities. The selection also considered age, gender, Aboriginal status, and region where participants had lived at the time of recruitment. All 241 people on the list were contacted by phone and invited to participate in the study. Of these, 50 people agreed to partake in the study and were further contacted to arrange the interviews.

Finally, 27 people agreed to be interviewed. The sample comprised eight EGM gamblers, eight sports and racetrack bettors, and 11 who had experienced harms from others’ gambling. Of 27 participants, 17 were male, the majority belonged to 35+ age group (*n* = 21), 20 were living in the Darwin-Palmerston region, and 17 identified themselves as non-Aboriginal (including three participants from culturally and linguistically diverse (CALD) background, i.e., those who did not speak ‘English only’ at home – this did not include any Aboriginal people) and 10 were Aboriginal (Table 1). Unlike quantitative studies, a sample of 5–50 is considered adequate for most qualitative research depending on the technique used [17, 18]. Culmination of the interviews also depend on “saturation” which occurs when adding more participants to the study does not result in additional perspectives or information [19]. In the current study, no new themes emerged after 25 interviews. However, we conducted two more interviews to confirm the saturation. The saturation was confirmed after 27 interviews and thus no further interviews were conducted.

**Table 1:** Demographic characteristics of the sample

Target group	Age		Sex		Region			Aboriginal status		Total
	< 35	35+	M	F	Darwin/ Palmerston	Alice Springs	Rest of NT	Aboriginal	Non-Aboriginal	
Affected others	3	8	4	7	6	4	1	4	7	11
Regular gamblers	2	11	10	3	11	0	2	3	10	13
Non-regular gamblers	1	2	3	0	3	0	0	3	0	3
Total	**6**	**21**	**17**	**10**	**20**	**4**	**3**	**10**	**17**	**27**

*M* Male, *F* Female

### Data collection

Based on relevant literature [5, 7] and input from relevant stakeholders (gambling researchers, gambling counsellors, etc.), separate interview guides were developed for each of the three categories of participants (see Additional File 1). There was a set of questions common across the guides, and a further set of questions that were specific to the individual guide, based on the participant category (e.g., type of gambling activity). Five to ten questions were developed for each objective. This approach ensured that the interviews provided a sufficient coverage of the topic.

Nearly all interviews were conducted by telephone (*n* = 25). Telephone interviews were chosen over face-to-face interviews because 1) they allow more anonymity to participants [20], 2) participants are more relaxed on the telephone, express their voices freely, and more likely to disclose intimate information [21], especially when the interview topic is of sensitive nature (gambling in this context), 3) they are cost-effective [22], and 4) allow a wide geographic coverage and access to geographically-distant subjects [23, 24]. Moreover, qualitative data gathered via telephone is considered equally robust and valid as face-to-face interviews [23, 24]. As the researcher (the interviewer) was based in Darwin, participants who were based in Darwin/Palmerston region were given a choice for face-to-face interviews. However, the majority of participants in the current study preferred phone interviews – because of the sensitive nature of the topic, they wanted to remain anonymous and thus expressed their voices freely over the telephone. Face-to-face interviews, where requested (*n* = 2), were conducted at a university office. All interviews were conducted by the first author of this paper, who was trained in mixed-methods research. For telephone interviews, the Participant Information Sheet (PIS) was read out to the participants and a verbal Informed consent was obtained at the beginning of each interview. For face-to-face interviews, participants read the PIS and signed the Consent Form prior to starting the interview. Each interview lasted for an average 30 min. All participants agreed for the interviews to be audio-recorded and notes were made by the interviewer during the interviews. All recordings were transcribed by a secure transcription service. Transcriptions were not returned to the participants for comments as we did not have the email IDs for majority of them. Participants were given a $50 grocery voucher for participating in the study. The voucher could not be used to purchase tobacco, alcohol, or gambling products.

Both semi-structured and structured questions were asked in the interviews. The semi-structured questions explored:

- negative impacts participants experienced from gambling (from own and others’ gambling);
- strategies used for controlling gambling;
- experience with help-seeking for gambling issues. This included probing on being approached by and talking with 1) personal contacts (e.g., family, friends, and work colleagues), and 2) formal services (e.g., GPs, counsellors, welfare organisations, etc.), about gambling; and
- strategies and interventions government and other agencies could use to minimise harms from gambling in the NT.

At the end of the interviews, gamblers were asked structured questions on the PGSI, to assess their problem gambling risk. It is a standardised nine-item scale for measuring the severity of gambling problems in the general population. The response choices for each PGSI item are ‘never,’ ‘sometimes,’ ‘most of the time,’ or ‘almost always,’ with a total score ranging from 0 to 27. Cut-offs used to assign gamblers to categories comprise ‘non-problem gamblers’ (PGSI = 0), ‘low-risk’ (PGSI = 1–2), ‘moderate-risk’ (PGSI = 3–7), or ‘problem-gambler’ (PGSI > 7) [25, 26]. Based on the PGSI scores, two participants in the current study were assigned to the ‘low-risk’, nine to the ‘moderate-risk’, and five to the ‘problem-gambler’ categories.

Participants were also given the opportunity to discuss anything they wanted about gambling that had not been covered during the interviews.

The Consolidated criteria for reporting qualitative research (COREQ) guidelines have been used for reporting this research - a completed COREQ checklist has been included as an additional file (see Additional File 2).

### Analysis

A Framework Analysis method was used to explore the interview data [27]. This method is flexible and has no allegiance to a particular theoretical approach and thus sits along the inductive-deductive continuum whereby themes from the literature and participant interviews are used to guide the analysis process [28]. Hence, this method (a) acknowledges the existing evidence-base; and (b) privileges consumer viewpoints as the primary point of reference [28, 29]. In this instance, we identified areas from the literature we wished to explore [5], but also wanted to discover the unexpected, that is, identify themes in our data.

NVivo 12 software was used to organise and manage the data. Both authors coded the data, resolved the disagreement in coding via discussions, and agreed on the final coding framework, hence establishing the credibility of the study’s findings. Participants are quoted throughout the results section. Sex (male and female), age group (18–24, 25–34, 35–44, 45–54, 55–64, and 65+ years), region (Darwin/Palmerston (D/P), Alice Springs (AS), and Rest of NT), Aboriginal status (Aboriginal and Non-Aboriginal), and participant category (affected others, EGM, and other betting) are noted for each quote. References to the PGSI scores are also made for gamblers.

### Ethics

Ethics approval to conduct this study was sought from the Central Australian Human Research Ethics Committee (CA-19-3310) and the Human Research Ethics Committee of the Northern Territory Department of Health and Menzies School of Health Research (2019–3294).

## Results

### Negative impacts experienced from gambling

Both gamblers and affected others reported having experienced negative impacts from gambling. Financial harm was the primary negative impact of gambling reported by many participants across groups. Other impacts were relationship conflicts, emotional distress, and decrements to health.

#### Negative impacts experienced from own gambling

Regardless of the cause and effect, participants described the circular associations between gambling, financial stress, and social and emotional wellbeing. Unsurprisingly, most participants in this cohort had high PGSI scores.

*You become distant. You won't really enjoy activities with friends if you don't have the money. You become isolated because you find gambling as your only form of addiction. It leads to stress and anxiety, yeah, and then you play even more.* (M, 25-34, D/P, Non-Aboriginal, PGSI 7, other betting)

Strong language such as “get a gun and shoot yourself” and “lose everything” were used by participants to indicate the severity of the losses:

*Sometimes when you put such an extreme amount of money on the pokies and then run out of money, then you realise what have you done. You know, like really if you could get a gun and shoot yourself. It just leaves you look like a stupid.* (M, 55-64, D/P, Non-Aboriginal, PGSI 6, EGM player)

This statement also draws attention to the shame and regret many gamblers often feel when they overspend on gambling. There were also participants who were thoughtful of their gambling and thus had not faced negative consequences. Ironically, one of such participants scored high on the PGSI:

*No. I just put a hundred dollars in my pocket and go [to the casino]. I don't keep any extra money with me, so if I win, I come back, if I lose I don't have any more money for playing.* (M, 25-34, D/P, Non-Aboriginal, PGSI 8, other betting)

#### Negative impacts experienced from others’ gambling

Participants in the affected others group were affected by their spouse’s gambling (*n* = 5), followed by a parent (*n* = 3), children (*n* = 1), friend (*n* = 1), and relative’s gambling (*n* = 1). They reported having experienced multiple harms from others’ gambling. The reported impacts were the struggle with finances, mental exhaustion, and difficulty in managing relationships, with varying degrees of severity:

*So, I'm 49 years old now. When I was 20 so this is nearly 30 years ago, and it still affects me now because it's still ongoing. I gave my mother all of my pay one day to pay the rent and do shopping. And when I returned home after work that day there was no shopping no food in the house and the rent hadn't been paid. So, we were nearly kicked out of our home Housing Commission home and we had to go to a relative's place to have dinner that night until I got some more money to keep on buying until the next payday. I didn't go out like I was always, I just wanted to stay home because I didn't have the money to spend anyway, because all my money was gone. So, it affected me that way where I couldn't go out and then I started saying to her [mother] that I didn't have any money because I didn't want to give her any money. So, I would tell her that I used it on something else.* (F, 45-54, D/P, Aboriginal, affected others)

There were instances where gambling had put stress on the relationships with spouse and children, usually in the forms of frustration and overstretching of the finances:

*It causes me frustration. We [my husband and I] are trying to make it like control our family budget. And whilst it's not huge amounts it still it still [sic] has an impact on my stress. It has affected our relationship to a minor degree. And just you know conflicts and discussions that I guess get a little more of an argument rather than a discussion. And and [sic] then [it] has a negative impact or overflow onto our children. They don't like hearing us argue that if it starts off as a discussion and then can quickly become into a bit more of an argument. So, voices have been raised but no physical violence. Whilst it is part of our family income, they're not they're not [sic] amounts that that [sic] would affect my ability to feed my children or pay my mortgage or my other bills. Just leaves us with less money for other sort of forms of entertainment.* (F, 35-44, Alice Springs, Aboriginal, affected others)

Apart from affecting families, gambling had also negatively impacted friendships and relationships with work colleagues:

*In the end I just told him [best friend] to please don't come near my door anymore because I'm not going to give you any money. Well it affected our relationship because I didn't want anything to do with him anymore. His wife and the kids ended up going into women's shelters because they had nowhere to live. He started asking for advance payment at work. And once I explained to his boss why he was doing it because his boss at that time was a friend of mine. And as far as his work colleagues go, they've all shunned him because he started asking them for money.* (M, 65+, D/P, Non-Aboriginal, affected others)

As a response to boredom and stress, participants from CALD background reported engaging with gambling activities, either themselves or their family members:

*Being in a different country with no family support and having arguments [because of gambling] with him [husband] is mentally very taxing and him losing money on gambling all the time is financially very draining.* (F, 35-44, D/P, Non-Aboriginal, affected others)

Intriguingly, none of the gamblers reported others having been affected by their gambling. However, a few of them refused to answer this question and were the participants with high PGSI scores:

*No, I don't think my gambling has affected anyone. My wife doesn't like it. but It's not to the point where I mean she just thinks it's pointless. But it doesn't cause any rift in our relationship or anything along those lines. I think [that’s] because we're not spending a huge amount of money on it anymore. She doesn't really like me going to the casino but I think it has got more to do with the beer than it has to be with gambling.* (M, 35-44, D/P, Non-Aboriginal, PGSI 5, other betting)

### Help-seeking for gambling issues and self-regulation strategies

#### Accessing help for gambling harms

Gamblers were asked to describe their experience with accessing help for gambling harms or issues they had experienced from their own gambling. The majority were aware of the existing support and services. However, most of them had not sought help or accessed services for gambling harms or issues.

*No, I've managed to pull myself up when I thought it was getting silly. But I'm aware of you know these anonymous hotlines. You can ring, those various websites so you can engage with. Yeah. If you know if you don't want to deal with a human being face to face or you can go face to face. There's thankfully for me because my father was a bad gambler. And you know I was able to observe that as I was growing up. I have. Yes, I have gambled and I still gamble in the ways I described. But I keep it under pretty tight control.* (M, 55-64, D/P, Non-Aboriginal, PGSI 5, other betting)

One participant described his experience with the support service he had used in the past as to how it had helped him to control his gambling in the long-term:

*I've been through a couple of sessions with a lady [counsellor]. The sessions went for about an hour. We were talking about how to better myself, what should I do, what shouldn't I do. That's how we went about it. And that was helpful. And it taught me a little bit. I don't gamble as much now as what I did.* (M, 45-54, D/P, Non-Aboriginal, PGSI 3, other betting)

Participants who were affected by others’ gambling were asked whether they sought help for themselves or offered help to those whose gambling affected them. The question generated varied responses. Some of them wanted to offer help but could not because the gambler had never admitted issues with their gambling.

*Even if I did he [son] wouldn't take it. He doesn't need help any sort of help that you offer for people if they're not willing to go themselves. Look I actually wanted him to go to my mindset coach that I use and get some adjustment to his attitude and his mindset and see if he would improve. But you know I wasn't fully aware of how bad it was. It's like he would talk about betting on the horses and he would only talk about when he wins, won't talk about the losses.* (F, 55-64, D/P, Non-Aboriginal, affected others)

In other cases, help was offered but was not accepted:

*No, never seek out [sic] any help for her [mother]. She always said that it wouldn't happen again, and 30 years later still till today I still help her now and again financially [sic] but she's still gambling. I always thought about it and you know picked up information you know being in my profession, I've picked up information along the way and thought about it. I think I did once or twice maybe slipped cards [self-help cards] into her purse and she got angry and upset with me for doing that. So, I never did it again.* (F, 45-54, D/P, Aboriginal, affected others)

#### Wanting help or support

The majority of the participants (gamblers) had never wanted help or support for any gambling harms or issues. The most popular reasons for not wanting or seeking help were 1) they had believed that they could deal with their problems themselves (keeping things private was a concern too) and 2) they thought their gambling was not bad enough to seek help:

*I wanted to but I never did. Because I wanted to keep it all private to myself.* (M, 25-34, D/P, Non-Aboriginal, PGSI 7, other betting)

*No, not at the moment. I'm not that bad. Yes, I know later on [sic] I want to stop. I know what my limits are.* (F, 55-64, D/P, Aboriginal, PGSI 4, EGM player)

Below participant was pleased with the support he had received; however, wished he could get more help:

*I could've got more sessions with this lady. She said after six sessions, there was nothing more she could help me with, but if she would ask me to come back, I would and talk about a bit more about my issue because that [the sessions] was helpful.”*(M, 45-54, D/P, Non-Aboriginal, PGSI 3, other betting)

One participant from CALD background wanted to seek help for her husband’s gambling issues, but being new to the country, she was not aware of the available support services. Moreover, she was hesitant to seek help because of the lack of awareness and understanding of western concepts of counselling and treatment:

*Being new to the country, I don’t know where to get help [from]. I also don’t know how it works here, because, like we are from a different culture, so these things may be different here, you know.* (F, 35-44, D/P, Non-Aboriginal, affected others)

### Self-help strategies

Self-help strategies were commonly used to limit rather than stop gambling. The reported strategies were 1) setting limits on spending, 2) taking set amounts of cash to venues, and 3) creating barriers to accessing money or limits on gambling expenditure.

*What I've done recently is self-control and limit my visiting to the pub and I have gone down to about fifty to sixty dollars for gambling. And it really helped me to regulate my gambling.* (M, 45-54, D/P, Non-Aboriginal, PGSI 4, other betting)

Other strategies included self-exclusion from venues and online betting services and using lines of credit. Many participants were aware of these strategies, but only a few had used them:

*And maybe you need to self-exclude or ask them the venues to ban you. I just sort of I have already did once this week already. I don't feel like it to go again [to venues].* (M, 55-64, D/P, Non-Aboriginal, PGSI 1, EGM player)

Some of them had found ways of consciously distracting their mind from gambling:

*Yeah I go out and do things that are different you know. I go out and be with people, talk to people, sit down and spend the day somewhere. It's when your mind is on it, you only think about it [gambling], it's all in the head.* (F, 55-64, D/P, Aboriginal, PGSI 4, EGM player)

*I keep myself busy cooking dinner instead of going to the club.* (M, 55-64, D/P, Non-Aboriginal, PGSI 1, EGM player)

Willpower was another tool used by some participants to resist the temptation to gamble:

*To me it was just like giving up smoking or giving up drinking. You just gotta [sic] be strong and sort of assure yourself that you ain't gonna [sic] do it. Just gotta [sic] just say no to yourself. You know what I tell myself that I am not gonna [sic] go out this week. I just got to live in the reality. I just have reduced it [gambling] quite a bit I haven't I haven't [sic] stopped altogether, but it helps.* (M, 55-64, D/P, Non-Aboriginal, PGSI 6, EGM player)

### Who is appropriate for gamblers to approach for help?

#### Within formal services

- By Counsellors

Many participants discussed the appropriateness of the counsellors (counselling psychologist, financial counsellor, etc.) being approached by gamblers for gambling issues. However, getting people to attend such a service in the first place was a major issue described by the participants:

*Maybe a psychological counsellor or financial services could help gamblers if they want to seek help. If someone goes out of their way to actually call up [the services], they have come to the realisation that they've got a problem and they've gone to the trouble of seeking help which is positive. Yeah, you've got to get that person to make that phone call in the first place.* (M, 55-64, D/P, Non-Aboriginal, PGSI 6, EGM player)

The cost associated with accessing such services was described as another barrier to seeking help:

*I guess psychologists or financial counsellors or someone in that sort of field. But a lot of time, a lot of financial involvement is there. Unfortunately, we couldn't really afford most of these. And in terms of getting them free, lot of them you get a very initial consultation and a little bit of guidance but anything more they require a payment or part payment and you just kind of go, Oh! I can't afford it.* (M, 35-44, D/P, Non-Aboriginal, affected others)

b) By General Practitioners (GPs)

Compared with the counsellors, approaching GPs for help-seeking did not appear to be an effective option:

*The services would, like I said Amity would or maybe a financial counsellor. But I think GPs can't, they don't specialise in gambling and you know to help with addictive behaviour like gambling you need someone that deals with this.* (F, 18-24, D/P, Non-Aboriginal, affected others)

However, GPs were suggested as possible points of referral to other, more specific services:

*I don’t think GPs could help in this case because they are not trained to do it, but they can tell you where to go for help.* (M, 25-34, D/P, Non-Aboriginal, PGSI 8, EGM player)

c) Other services

Although welfare services such as Salvation Army and Centrelink were discussed in a positive light in the context of help-seeking, not many participants were aware of such services. Further, attending services in person was difficult for some participants due to the shame and stigma associated with gambling. Hence, they preferred seeking help from services such as Gamblers Anonymous (GA) and/or a phone chat, to maintain the anonymity:

*Like we have Alcohol Anonymous for people with alcohol problems, where they can talk about their issues but no one can see them, and there is no shame or no one call you bad, likewise I would go to Gamblers Anonymous because of the anonymity. Or I would like to talk someone on phone like I’m talking to you [the interviewer] right now.* (M, 25-34, D/P, Non-Aboriginal, PGSI 7, other betting)

Some participants emphasised the importance of peer-support in this context:

*I reckon again if you go with someone, it will really be helpful in this sense that if you keep on losing and that person understands their situation then they'll stop you. Otherwise you will keep putting in the money. So, it’s good to have a company and it could be helpful.* (M, 55-64, D/P, Non-Aboriginal, PGSI 5, other betting)

#### By personal contacts

- By family and partners

The role of the family was discussed in a positive light for help-seeking, across interviews. The main factors associated with approaching the family were trust and comfort between family members. Seeking help from partners was also discussed; however, to a lesser extent:

*I believe that family would definitely be helpful because it’s the first line of defence, but not [work] colleague because if you're at work, you don't really want your whole workplace know that you're a gambler.* (F, 18-24, D/P, Non-Aboriginal, affected others)

*Yeah. It depends on your support network around you I suppose. So, but to me you know because if I if I [sic] had a problem with gambling and I really wanted to do something about it then by all means I don't think I'd go to my wife because she doesn't like it [gambling]. But It's not to the point where I mean she just thinks it's pointless. But you know there would be friends and family around me that I could sit with and speak to about it.* (M, 35-44, D/P, Non-Aboriginal, PGSI 5, other betting)

Barriers to reaching out to the family for help were 1) a lack of self-realisation of gambling issues and 2) bad relationships with family members:

*Maybe family members if the gambler thinks it's a problem which often I think in my experience it's not. They don't actually think that they have a problem. In my experience with my husband if he gambles at the casino and if he has a big win, he just wants to gamble more. He sees it just a way to make money but he doesn’t realise the risk of losing money that he has just won.* (F, 35-44, Alice Springs, Aboriginal, affected others)

*When I say that I don't have family btw, I do have mother and father but I don't see them. So, I wouldn't approach them. I would approach nobody other than as I've done recently is self-control and limit my visiting to the pub and I have gone down to about fifty to sixty dollars for gambling. And it really helped me to regulate my gambling.* (M, 45-54, D/P, Non-Aboriginal, PGSI 4, other betting)

b) Through friends and work colleagues

After family, friends were the preferred choice for gamblers to discuss gambling problems with. However, shame and embarrassment were discussed as barriers to approaching friends in some cases:

*It's definitely appropriate for gamblers to reach out to friends or their work colleagues or whoever they can, to seek help. That step is going to take you to getting help to tell people about it.* (M, 25-34, Rest of NT, Non-Aboriginal, affected others)

*In terms of friendships or whatever it was it's it's [sic] not something that we've really discussed with too many people because I think she's [wife] dealing with the you know [sic] she’s very embarrassed and very ashamed [of her gambling issues], she's admitted to it and she keeps talking to me about it.* (M, 35-44, D/P, Non-Aboriginal, affected others)

Compared with family and friends, work colleagues were deemed appropriate for help-seeking, to a much lesser extent. The main reason for this was stigma attached with gambling. One participant specifically described the need for destigmatising gambling so that gamblers can readily approach friends and work colleagues for help:

*For me I did talk to one of my work colleagues about it [wife’s gambling issue] in a very general sort of manner. She's pretty much only told I think three or four people about it because she's very embarrassed and very ashamed, even now, more than 12 months on [sic]. For me it was good just to kind of see someone else's opinion. The person I talked to I wouldn't say they had a gambling problem but they put a few bets here or there and they go to the pokies every few weeks and I thought maybe they'd have a bit of a different perspective on things. That was kind of helpful.* (M, 35-44, D/P, Non-Aboriginal, affected others)

*I do think it is appropriate for gamblers to approach friends or maybe work colleagues to seek help if they see that they have problem gambling. Yeah, I think that there should be enough information out there for a friend or a colleague to be able to refer a friend or colleague to a gambling service. But it is not something that I believe in. It should be destigmatised to a point where a colleague or friend can refer to a gambling organisation but it doesn't have to be anybody or everybody's business.* (F, 45-54, Alice Springs, Aboriginal, affected others)

c) In venues

Some participants discussed about the resources available at the venues (such as gambling helplines and posters put up at the back doors of the toilets) for gamblers to access for help:

*You know there's lots of posters and signs in the gambling room saying - if you gamble too much, seek help. I think honestly being on the back of a toilet door is more effective because they've got to go to the toilet at some point and having the anti-gambling signs is more effective on the back of a toilet door than in the pokies room [sic].* (F, 18-24, D/P, Non-Aboriginal, affected others)

*Well there are services available on every pokie machine you've got a number to call if you are having trouble with gambling. I can't tell you the number because I've never used that.* (M, 65+, Rest of NT, Non-Aboriginal, PGSI 4, other betting)

d) Others

Participants also described other resources that were available for gamblers to access for gambling issues. The resources included 1) web-based applications that help people to monitor their gambling 2) and online and offline (e.g., television) advertisements containing information on gambling helplines:

*I guess since all those gambling helplines that pops up on TV or on [mobile] phone or you can probably Google gambling helplines or something for gamblers. I guess on the Internet these days you can get any information.* (F, 35-44, Alice Springs, Aboriginal, affected others)

Online assistance was preferred over other sources, especially where there were issues of embarrassment and shame associated with gambling:

*So, had I not done that I probably would have kept going. But in general terms it's very hard because people don't want to talk to someone even having a phone server you're still talking to someone. Perhaps Internet where they can remain anonymous. Like I said my wife she underwent a lot of shame and felt very very embarrassed for quite a while after it so I doubt she'd want to have identified herself publicly in any way. So, I guess some anonymous sort of service would probably be a way forward.* (M, 35-44, D/P, Non-Aboriginal, affected others)

### Measures to minimise harms from gambling

Participants were asked for their views about what they thought government could do to minimise harms from gambling in the NT. The discussions primarily focused on seeking opinions on preventive measures rather than on post-harm interventions.

Participants strongly recommended for the government to make stricter legislation around gambling. However, they were aware of the revenues government, venues, and the gambling industry generated from gambling, and thus were unsure of the actions government might exercise to regulate gambling:

*I think they [revenues from gambling] are necessary evil for the government because of the tax or the revenue that they get out of it. So, it's not in their best interests to provide funding for helpline and counselling because they don't care to stop people from gambling because they get so much out of it. You know it's a really grey area.* (M, 35-44, D/P, Non-Aboriginal, PGSI 5, other betting)

The suggestions included restricting the daily number of hours of playing and the bet size, and limiting the number of EGM at the venues:

*Perhaps minimise the number of machines, minimise the amount of margins venues get, and minimise the amount of bet, so, you know the maximum bet becomes a dollar rather than 10 dollars or so. And like I said minimise the amount you can win.* (M, 35-44, D/P, Non-Aboriginal, affected other)

*I wouldn't mind if they [government] scrap them [pokies]. But it's too much revenue from them they'll never ever do it. So, if they can limit how much a person can bet in one go would probably be the best. But they won’t because like I said they don't want to.* (M, 65+, D/P, Non-Aboriginal, PGSI 12, EGM gambler)

The potential effectiveness of implementing pre-commitment cards to limit the bet size was also discussed:

*Some policies can be made like after a certain amount of money you spend on gambling, you can't do anymore. Setting a limit, you know, so that it doesn't cause any financial and mental stress to the gamblers’ family. The person can only play up to this limit.* (F, 45-54, D/P, Aboriginal, affected other)

*That'd be good if they [government] could limit the amount that you spend on them [pokies] in the venues on a given day and you can use only some kind of gambling card to load the money on and play. So, once you’ve reached that limit, you can’t play anymore and wait 24 hours for the next play. The system should apply throughout the state doesn't matter where you go. If you’ve reached the limit and want to use the card in the venue they will say oh you've already spent the maximum amount for the day and you can't spend anymore. You know something like that.* (M, 65+, D/P, Non-Aboriginal, PGSI 12, EGM gambler)

This was thought of as an effort towards reducing the amount of gambling and the revenues:

*Reducing the number of machines is a big place to start. Casinos I think they are an entertainment venue. I don't know whether we need lots and lots of pokie machines in there [community venues].* (M, 35-44, D/P, Non-Aboriginal, PGSI 5, other betting)

*I think you wouldn't be chasing such big jackpots either. I think that's part of the problem with my wife as she lost some money and she tried to win it and she lost some more and lost some more and then her betting got significantly bigger because you don't win on small bets you only win on big bets allegedly, so, you know, reducing the revenue to the owners of the machine I guess would help [sic].* (M, 35-44, D/P, Non-Aboriginal, affected other)

Limiting the opening hours of the venues was described as another potential measure to reducing the amount of gambling:

*Yes, definitely, the hours are terrible. I think the gaming rooms open at 8:00 or 9:00 in the morning and then they close at 3:00 a.m. You know they kind of carry on. The longer they are open, the more opportunities for people to sit down. You know just reduce the number of hours that they can operate and the impact will be far more. They are just open all the time.* (M, 35-44, D/P, Non-Aboriginal, PGSI 5, other betting)

Adapting successful international gambling legislation in the Australian context was also suggested by one participant:

*I think in Singapore the local people aren't allowed to go to the casino, they have to pay to go there or something like. So, if that were the case here [in Australia], it might stop people from going if they had to pay an entry fee. And if they don't allow membership of clubs at casinos because they are an incentive to keep going, it would prevent people to go there.* (M, 55-64, D/P, Non-Aboriginal, PGSI 5, EGM gambler)

The need for creating advertisements containing powerful messages about gambling-related harms was also stressed:

*I think government could make advertisements wherein there are messages from gamblers on how it [gambling] has affected their lives and health in terms of stress and stuff like that. So, people get to know that how gambling affects other people in gamblers’ lives and not just the person who is actually doing it. These kinds of ads will be much more effective than the regular ads, you know.* (F, 35-44, Alice Springs, Aboriginal, affected other)

Regulating the timing of the advertisements was also discussed:

*So, I think online betting should be treated like alcohol and tobacco. I think my nephew and his girlfriend both do online gambling as well. And I think it is remarkably addictive for them. I think the advertising on TV should be like tobacco and alcohol and should be banned like no advertising before 9 pm. And I think it should be an integrated approach.* (M, 55-64, D/P, Non-Aboriginal, affected other)

Participants also recommended these efforts to be targeted at kids and younger people, as a preventive measure:

*I think it all begins with education at a very early age in the schools. That's where we learn everything… you know… the negatives. The kids should be told about the negative effects of gambling before they get to know it in the adulthood, like for most other things including alcohol.* (M, 55-64, D/P, Aboriginal, PGSI 2, other betting)

Participants were asked their opinions about the change in policy that allowed note acceptors to be installed on EGM located in hotels and clubs, and people to load up to $1000 in any denomination of note into the EGM instead of the previous dollar coin system. Most of them described the negative implications of note acceptors in EGM and advocated for re-introducing the coin system:

*There's a lot of difference between one dollar and one thousand dollars. People who have less money play with like ten dollars, 20 or 30 dollars, but if they earn like thousand dollars they will play with hundred dollars and keep on playing until they lose like ten thousand dollars. If they put like one dollar and lose one dollar, they end up losing like 20-30 dollars, which will make a big difference [to their losses].* (M, 25-34, D/P, Non-Aboriginal, PGSI 8, EGM gambler)

*Yeah, that [the dollar coin system] would slow it down. You know the note acceptors and I've seen people put in 50 after 50 after 50. And for example, if they were to put in 200 coins it would take them some time to reach that dollar amount.* (M, 55-64, D/P, Non-Aboriginal, PGSI 1, EGM gambler)

Participants discussed the easy accessibility of online gambling and the potential associated harms, and thus stressed the need for a stronger regulation:

*I think there should be stricter laws made on especially online gambling. I think it's very accessible for people of any age to be able to transfer money from… you know… with internet banking being so accessible these days people being able to transfer money into online accounts. I think this needs to be restricted.* (F, 35-44, Alice Springs, Aboriginal, affected other)

However, regulating online gambling was considered ‘tricky’ by some participants. The need for finding alternative ways of entertainment was also discussed:

*I'm not someone that would support online gambling simply because it's not the right environment for people to be trying to appreciate gaming. Even people who are incapacitated or injured are able to do it. So, the accessibility should be reduced. There should be certain limits set on how much a person can gamble and there should be a better entertainment instead. But then what all would you regulate, everything can be done through back door, you know.* (M, 55-64, D/P, Non-Aboriginal, affected other)

Strong views against online gambling were also expressed:

*They [online gambling] are a lot cheaper than the pokies and should not be allowed at all in Australia.* (M, 25-34, Rest of NT, Non-Aboriginal, affected other)

Participants appeared to be unsure of bringing in gambling-related issues with gamblers in normal conversations. Therefore, the necessity for developing resources to help them with the issue was voiced:

*I think I think [sic] I've said at the start that this gambling issue for my mother… it has been 30 years so far. So, hopefully you know if there is some more literature or education or things that can be done by the government to help people like myself to start a conversation with the gamblers and how to stop them or help them to stop. That would be a big help. You know because at the moment it's still very hard to try and tell somebody when they think that they don’t have a problem.* (F, 45-54, D/P, Aboriginal, affected other)

## Discussion

This study is the first in-depth qualitative study of gambling that has been conducted with a purposeful sample of regular gamblers and affected others in the NT. It provides insights into the experience of individuals who reported experiencing gambling-related harms, help-seeking for gambling issues, and their views on current legislation on gambling. In terms of negative impacts experienced from own gambling [5, 7] and from others’ gambling [6], help-seeking for gambling issues [5, 7], and self-help strategies used for controlling own gambling [5, 7], our study generated similar results to those identified in previous studies conducted in other Australian jurisdictions. However, what distinguishes this study from the other studies is 1) we interviewed both gamblers and those affected by someone else’ gambling, 2) the sample comprised both Aboriginal and Non-Aboriginal (including people from CALD background) people, 3) the sample had representation from both urban and remote areas of NT (more participants from urban areas), 4) participants’ experiences with help-seeking in multiple contexts (personal contacts, formal services, and third parties), and 5) participants’ opinions on gambling legislation in the NT, were explored. Hence, having been able to capture the experiences of a variety of voices is one of the strengths of this study.

Previous qualitative studies conducted in the NT had a different focus from our study in that they explored gambling activities (less about commercially available gambling and more about cards) and related harms (especially, around problem gambling) in a few remote Aboriginal communities [13, 14].

Overall, the study’s findings help to gain a greater understanding of the lived experience of gamblers and affected others, complimenting the existing quantitative gambling research in the NT [9]. Viewing the findings from a public health lens, both a community-wide and targeted approach will have the potential to address and minimise harms from gambling. The approach needs to be inclusive of the experiences and understandings of people who are at risk of gambling harm (preventive approach) or are experiencing it (interventional approach) [5, 30–32], that is, tailored to address the causes and impacts of harms experienced by people [5, 6].

This study has highlighted that those with lived experiences of gambling have identified a range of strategies to reduce harms from gambling through community education, pre-treatment approaches, commitment technology, and gambling legislation to reduce harm from gambling in the NT.

### Community education

A lack of self-awareness of gambling behaviour was identified as one of the barriers to control own gambling and thus help-seeking, with stigma and shame both contributing to gamblers and affected others not seeking help.

Gamblers, who were aware of their gambling issues, were also resistant to seeking professional help, especially the high-risk gamblers. Initiatives that portray positive outcomes from gambling interventions may encourage them to seek help. For example, creating advertisements featuring gamblers who had previously benefitted from gambling interventions. Further, interventions such as supporting the use and success of self-help and self-regulation strategies are important for these individuals. Low- and moderate-risk gamblers in our study were more receptive of the negative impacts and harm from own gambling than high-risk gamblers. Subsequently, some of the low- and moderate-risk gamblers sought help which was primarily via informal sources and mainly comprised self-help strategies. On the other hand, many high-risk group gamblers had not sought any form of help because of a lack of acceptance of their gambling issues. Low-and moderate-risk gamblers make up the majority of at-risk gamblers, so preventive interventions targeted at these groups may have a significant impact in addressing and minimising absolute levels of gambling harm in the community. The strategies aimed at improving awareness of gambling behaviour such as keeping track of losses and venues issuing regular statements on gambling expenditure to their patrons may facilitate the self-identification of gambling issues [33–35].

Public education campaigns aimed at reducing the stigma associated with problem gambling and being harmed by someone else’s gambling may be helpful in improving uptake to help services. For example, Miller and Thomas [36] found that ‘responsible gambling’ discourses contributed to felt and enacted stigma associated with problem gambling by focussing on personal responsibility, and that they could lead to personal blame and shame, and further contributed to negative stereotypes. Sustained public education campaigns aimed at reducing stigma associated with gambling problems, promoting uptake of services, and showing success in treatment should be considered by governments.

Many deemed personal contacts appropriate to approach for help and vice-versa. Involving the support of others in behavioural strategies was helpful in controlling own gambling. Interventions such as “The Stress-Strain-Coping-Support Model” [37] and the “5-Step Brief Intervention” [38] have been successfully adapted in the gambling context to support family members (affected others) of people with gambling issues to cope with the harms. However, interventions that focus on assisting affected others to help the gamblers regulate their gambling and thus minimising associated harms, are lacking. This highlights the need for interventions supporting affected others (such as family and friends) to help protect their wellbeing and also helping and motivating non-help seeking gamblers to acknowledge their problem and seek help [39]. There has been some work in this space. For instance, Bond et al. (2017) conducted a Delphi study with a range of people working in services treating mental health and gambling problems, which developed guidelines and strategies to assist Aboriginal affected others to discuss gambling and help-seeking options with their close ones experiencing gambling-related problems [40, 41]. An evaluation of this study found that people who did use it found it helpful when discussing gambling issues with Aboriginal gamblers [42].

### Training for allied health services

Many participants raised the issue of a lack of training among health professionals and service providers to support people experiencing harms from gambling. Therefore, interventions that encourage professionals to raise gambling with their clients and conduct brief interventions may have potential in reaching people experiencing difficulties. Moreover, the mental health referral can be used as a pathway where a GP can refer people with issues to a psychologist for Medicare covered services. Also, as suggested by participants, rather than focusing on number of sessions with the psychologist/counsellor, longer and more spread out sessions (such as over 6–12 months) sessions may be needed.

### Stronger regulation and legislation

Regular gamblers indicated that they were unaware of their gambling behaviours and how they may influence their own gambling problems. Therefore, strategies aimed at improving awareness of gambling behaviour such as keeping track of losses and venues issuing regular statements on gambling expenditure to their patrons may facilitate the self-identification of gambling issues.

Various studies on pre-commitment have found mixed results on effectiveness. For example, research conducted with 30 regular EGM gamblers found that many had positive attitudes towards such a system, but that they saw it as more relevant to those experiencing problem gambling, and it would need to be flexible and customisable and easy to use [33]. However, mandatory pre-commitment does have potential, with a study in Germany showing that slot-machine gamblers who pre-commit to spending limits gambled less frequently and had a lower weekly spend, compared with gamblers not pre-committing to money limits [34].

Participants also appeared to be wary of the easy accessibility of online gambling and the potential associated harms. Thus, there is a need for a stronger regulation limiting accessibility to and setting mandatory load up limits on, online gambling, especially for younger people. Pre-commitment can also be applied to online gambling platforms. Some Australian gambling online platforms include a number of similar type tools to assist gamblers in controlling their gambling, though research found that there was limited uptake among gamblers (less than 25%), though uptake was higher among gamblers at higher risk of problem gambling [35]. These types of measures clearly need to be mandatory and not opt in.

The government and gambling industry position as regulators and receivers of money from gambling was acknowledged by participants of the study. Participants had a number of suggestions for stronger and more protective regulation of EGM and included, (i) setting limits on the daily number of hours of EGM playing, (ii) maximum bet size allowable (and via introducing pre-commitment cards), (iii) setting lower load up limits and related to this re-introducing the coin system for EGM and (iv) reducing access to EGM through reduced hours of access to EGM in community venues, has potential to limit gambling and gambling-related harms in the community. Hence, exploring the conflict between government and the gambling industry as both regulators and benefactors of gambling is another area of gambling research.

In 2013, the NT Government changed EGM policy on load-up limit into machines, stating that there was no evidence to indicate that this would lead to higher levels of problem gambling. However, research overseas had found that limiting load-up of EGM type machines was one of the single biggest policy change governments could do to reduce harm associated with EGM [43].

Further, a study published in 2019 looked at the effect of the change in load-up on EGM losses in the NT, and found strong evidence that the change in load-up led to increases in losses for EGM gamblers experiencing problem gambling [44]. In the 4 years following the installation of note acceptors on to NT EGM, user losses increased in community venues from $65 million to $96 million (more than 50% increase), while in preceding years user losses had been steady or in decline [26]. It would seem that governments are using this change in policy for no other reason than to increase their tax revenue intake, at the expense of EGM gamblers and those around them that are harmed by their gambling. Disappointingly, the South Australian Government did a virtual identical press release in 2019 about a change from coin to note acceptors to their EGM in community venues, again stating the there was no evidence that it would lead to more harm.

### Limitations and future research

Although the final sample was smaller than what was planned, the theoretical principle of saturation was reached [45], where new findings or insights were no longer revealed during the interviews. Therefore, interviewing more people would not have necessarily generated much new or relevant information.

The study was confined to residents of the NT and their gambling experiences. Hence, caution must be exercised when generalising these findings to other locations and the wider community. Nonetheless, as occurs with qualitative research, our aim was not to generalise the study’s findings to the wider community, but to provide an overview of the studied phenomena.

Similar responses were received from Aboriginal and Non-Aboriginal participants on questions relating to harms and help-seeking. This is likely because, in general, 1) gambling-related attitudes, beliefs, and expectancies develop through repeated exposure and conditioning [46]; 2) most of the harms from gambling are generic [47]; and 3) the associated shame and embarrassment are universal [48, 49], irrespective of the demographic group.

However, exploring people’s opinions and experiences from a cultural perspective is likely to generate different responses. Hence, future research might consider exploring people’s perspectives from a cultural lens, preferably, studying different population groups in separate studies. For example, a lack of understanding and awareness of western concepts of counselling and treatment, and awareness of the available support services was a potential deterrent for participants from CALD background to seek help. Given an estimated 100% increase in CALD population between 2006 and 2016 [50] in the NT, and that people from CALD background are at higher risk of problem gambling in the NT [51], it would be worth exploring diverse range of opinions and attitudes to gambling, recognising that not all individuals and communities share the same views or values, in future studies. This will help to design culturally and linguistically appropriate gambling support services and minimise harm from gambling among CALD communities in the NT.

## Conclusions

From a public health perspective, the study’s findings highlight the need for targeted and tailored treatment, policy, and regulatory approaches to address the causes and impacts of gambling-related harms experienced by people in the NT.

## Supplementary Information

**Additional file 1:.** Interview schedules. This file contains interview schedules that have been used for data collection.

**Additional File 2:.** COREQ Checklist. This file contains a completed COREQ checklist that has been used for reporting this research.

## Data Availability

The datasets used and/or analysed during the current study are available from the corresponding author on reasonable request.

## Abbreviations

CALD: Culturally and Linguistically Diverse
COREQ: Consolidated criteria for Reporting Qualitative Research
EGM: Electronic Gambling Machines
NT: Northern Territory
PGSI: Problem Gambling Severity Index

## Footnotes

1 For the convenience of writing, participants were categorised into two groups - gamblers and people negatively affected by someone else’s gambling (i.e., affected others), and referred to as such, throughout this paper.
