# Supplementary material for: “It started 30 years ago, and it still haunts me”: an exploratory investigation of Territorians’ gambling behaviours, harm, and help-seeking for gambling issues in an Australian jurisdiction
Source: BMC Public Health. 2021 Jan 7;21:96. doi: 10.1186/s12889-020-10141-5 (PMC7791886; doi:10.1186/s12889-020-10141-5)
Supplement: Supplementary file 1 — Additional file 1:. Interview schedules. This file contains interview schedules that have been used for data collection. [file 12889_2020_10141_MOESM1_ESM.docx]

### ADDITIONAL FILE 1: INTERVIEW SCHEDULES

#### ***Interview schedule - EGM gamblers***

**1. Gambling behaviour**

- Tell me about your gambling activities – type of activities, how often do you play, money spent on pokies, where do you play (gambling locations), when (cover current and past), wins/losses, etc.
- Why do you gamble?
- Did you go with anyone?
- How did you use venue (what else did you do in the space)?

(probe for having access to smoking room, alcohol, cash through eftpos and ATM withdrawals at the venue)

**2. Gambling problems and being personally approached**

- What you think how regular gambling can lead to other problems?
- Tell me about the negative impacts you experienced from your own regular gambling.
- What are your experiences of others around you (family, friends, work colleagues, etc.) being affected by your gambling. In what ways did it affect them?
- Have you been approached by venue staff?
- Were you gambling at the time?
- What were the reasons they approached you for?
- How did you feel being approached about gambling (not necessarily problems) by venue staff - was it appropriate?
- Did they offer any information, resources or services? If so, please tell me about them. Were they helpful for you? What parts were helpful? What parts were not so helpful?
- Other than venue staff, have you ever been approached about gambling (not necessarily problems) by anyone else - was it appropriate?
- Who, what did they say, how did they go about it, were you gambling at the time, in a venue. Was it ok?

**3. Who is appropriate to approach people about gambling (not necessarily problems)?**

I’m going to ask you a few more questions….

- Who do you think might be appropriate for gamblers to approach about their gambling? This might include offering help or assistance with help-seeking for gambling problems (other than venue staff).
- Separately ask about:
- partner, other family
- friends,
- colleagues
- Services (e.g. GPs, health services, counsellors, welfare, financial counsellors)
- Anyone else?
- Are any of these inappropriate?

**4. Who would be the best to approach - someone, how, where, when?**

One of the things we want to find out is how to best approach someone about their gambling, so things might not get so bad. We know some people can find it uncomfortable to approach someone to help.

- Who do you think would be best placed to help people ‘so things don’t get so bad’?
- What do you think would be best to say?
- In a venue?
- While gambling?
- How bad would things need to be for you to say something?
- What do you think might encourage people to get help?
- Do you think there is a way that would make you/other people feel more comfortable talking about gambling?

**5. Other strategies and interventions**

- Did you use any strategies to control gambling?
- What kind of strategies did you use? (also ask about self-help, self-exclusion from venues and online betting services, used lines of credit)
- How did they help you to regulate your gambling?
- In May 2013, the NTG legislated to allow for the installation of note acceptors on EGMs in clubs and pubs, allowing gamblers to load up to $1000 in any denomination of note. Tasmania and SA only allow $1 coins to be inserted (same as NT before change). The change in the NT has seen a large increase in EGM player losses.
- Do you think it would make EGMs less harmful (e.g. spend less money) if people could only insert coins?
- How much money do you think someone should be able to put into an EGM in one go?
- Think about the EGM venue/s you usually go to
- Do you have access to cash through eftpos and ATM withdrawals at the venue?
- What do you think could be the consequences of handling cash while gambling (both positive and negative)?
- *Prompt: ease of playing, higher risks of robbery.*
- Others - reduced hours of playing, reduced bet size, etc.?
- Pokies often display lighting effects, jackpot messages, good fortune messages and melodies? How do you think they affected your gambling behaviour?

**6. Help-seeking and health, recent and past**

- Have you ever wanted/got help for gambling problems?
- What were the reasons for seeking help?
- Who did you talk to for assistance in finding a gambling help service? OR How did you find a gambling help service?
- What kind of help you wanted?
- Did you get what they wanted?
- Was there any assistance you might have wanted that you didn’t get?
- I am going to ask some questions about health and services. I would like you to think about when you’ve been gambling a lot, were you seeing
- GPs;
- health services;
- counsellors;
- welfare services;
- financial services;
- any other?

***Ask at the end of the interview -*** What do you think governments could do to minimise harms from gambling?

***PGSI***

We will also ask a handful of questions we asked in the previous phone interviews. (For face-to-face interviews, offer to ask them the PGSI questions or fill it in and put it in an envelope (confidential)).

Now I’d like you to think about all your gambling in the past 12 months...

INTERVIEWER NOTE: PLEASE READ OUT "thinking about the last 12 months" READ OUT BEFORE EVERY STATEMENT

| DO NOT FLIP – KEEP GRID IN ORDER | Never | Some times | Most of the time | Almost always |
| --- | --- | --- | --- | --- |
| PGSI1 - Thinking about the past 12 months, how often have you bet more than you could really afford to lose? Would you say: (1) | 0 | 1 | 2 | 3 |
| PGSI2 - Thinking about the past 12 months, how often have you needed to gamble with larger amounts of money to get the same feeling of excitement? (2) | 0 | 1 | 2 | 3 |
| PGSI3 - Thinking about the past 12 months, how often have you gone back another day to try to win back the money you lost? (3) | 0 | 1 | 2 | 3 |
| PGSI4 - Thinking about the past 12 months, how often have you borrowed money or sold anything to get money to gamble? (4) | 0 | 1 | 2 | 3 |
| PGSI5 - Thinking about the past 12 months, how often have you felt that you might have a problem with gambling? (5) | 0 | 1 | 2 | 3 |
| PGSI6 - Thinking about the past 12 months, how often have people criticized your betting or told you that you had a gambling problem, regardless of whether or not you thought it was true? (6) | 0 | 1 | 2 | 3 |
| PGSI7 - Thinking about the past 12 months, how often have you felt guilty about the way you gamble, or what happens when you gamble? (7) | 0 | 1 | 2 | 3 |
| PGSI8 - Thinking about the past 12 months, how often has gambling caused you any health problems, including stress or anxiety? (8) | 0 | 1 | 2 | 3 |
| PGSI9 - Thinking about the past 12 months, how often has your gambling caused any financial problems for you or your household? (9) | 0 | 1 | 2 | 3 |

CPGI_SCORE = PGSI1 + PGSI2 + PGSI3 + PGSI4 + PGSI5 + PGSI6 + PGSI7 + PGSI8 + PGSI9

#### ***Interview Schedule - Other bettors***

**1. Gambling behaviour**

- Ask about gambling activities –
- type of activities: sport like AFL, cricket or tennis (excluding fantasy sports or footy tipping competitions), horse or harness or greyhound racing (excluding sweeps)
- how often do you play?
- money spent
- where do you play (gambling locations – online, sports company, app, pub, club, casino, over the telephone, other)
- when (cover current and past)
- wins/losses, etc.
- Why do you gamble?
- Did you go with anyone (for in-venue sports/racetrack betting)?

**2. Gambling problems and being personally approached**

- What you think how regular gambling can lead to other problems?
- Tell us about the negative impacts you experienced from your own regular gambling.
- What are your experiences of others around you (family, friends, work colleagues, etc.) being affected by your gambling. In what ways did it affect them?
- Have you been approached by betting company (online gamblers)?
- Were you gambling at the time?
- What were the reasons they approached you for?
- How did you feel being approached about gambling (not necessarily problems) - was it appropriate?
- Did they offer any information, resources or services? If so, please tell me about them. Were they helpful for you? What parts were helpful? What parts were not so helpful?
- Other than the betting company, have you ever been approached about gambling (not necessarily problems) by anyone else - was it appropriate?
- Who, what did they say, how did they go about it, were you gambling at the time, in a venue. Was it ok?

**3. Who is appropriate to approach people about gambling (not necessarily problems)?**

I’m going to ask you a few more questions….

- Who do you think might be appropriate to approach people about their gambling? This might include offering help or assistance with help-seeking for gambling problems (other than the betting company).
- Separately ask about:
- partner, other family
- friends,
- colleagues
- Services (e.g. GPs, health services, counsellors, welfare, financial counsellors)
- Anyone else?
- Are any of these inappropriate?

**4. Who would be the best to approach - someone, how, where, when?**

One of the things we want to find out is how to best approach someone about their gambling, so things might not get so bad. We know some people can find it uncomfortable to approach someone to help.

- Who do you think would be best placed to help people ‘so things don’t get so bad’?
- What do you think would be best to say?
- In a venue?
- While gambling?
- How bad would things need to be for you to say something?
- What do you think might encourage people to get help?
- Do you think there is a way that would make you/other people feel more comfortable talking about gambling?

**5. Other strategies and interventions**

- Did you use any strategies to control gambling?
- What kind of strategies did you use? (also ask about self-help, self-exclusion from venues and online betting services)
- How did they help you to regulate your gambling?
- Since February 2018, online wagering providers have been prohibited from offering any credit, voucher, reward, or other benefit as an incentive to open an account, to people who gamble on their site or app, or to refer another person to open an account.
- Do you think the government should change legislation to go back to previous measure wherein online wagering providers online would act like a bank to offer lines of credit to online gamblers? If so, why?
- How do you think this measure would help to protect consumers?

*Prompt: This measure is designed to protect consumers from incentive-based marketing and strengthen standards for direct marketing.*

*Prompt: It also includes preventing turnover requirements to withdrawing winnings from complementary betting credits or tokens.*

- Online wagering providers are required to use the same messaging about the risks and potential harm of gambling in their advertising, direct marketing, websites, and other direct communications to their customers.
- How do you think this measure would help to protect consumers?

*Prompt: Would this help to avoid inconsistent or ineffective messages about responsible gambling, and make sure messages reach people as they are making gambling decisions?*

**6. Help-seeking and health, recent and past**

- Have you ever wanted/got help for gambling problems?
- What were the reasons for seeking help?
- Who did you talk to for assistance in finding a gambling help service? OR How did you find a gambling help service?
- What kind of help you wanted?
- Did you get what they wanted?
- Was there any assistance you might have wanted that you didn’t get?
- I am going to ask some questions about health and services. I would like you to think about when you’ve been gambling a lot, were you seeing
- GPs;
- health services;
- counsellors;
- welfare services;
- financial services;
- any other?

***Ask at the end of the interview -*** What do you think governments could do to minimise harms from gambling?

***PGSI***

We will also ask a handful of questions we asked in the previous phone interviews. (For face-to-face interviews, offer to ask them the PGSI questions or fill it in and put it in an envelope (confidential)).

Now I’d like you to think about all your gambling in the past 12 months...

INTERVIEWER NOTE: PLEASE READ OUT "thinking about the last 12 months" READ OUT BEFORE EVERY STATEMENT

| DO NOT FLIP – KEEP GRID IN ORDER | Never | Some times | Most of the time | Almost always |
| --- | --- | --- | --- | --- |
| PGSI1 - Thinking about the past 12 months, how often have you bet more than you could really afford to lose? Would you say: (1) | 0 | 1 | 2 | 3 |
| PGSI2 - Thinking about the past 12 months, how often have you needed to gamble with larger amounts of money to get the same feeling of excitement? (2) | 0 | 1 | 2 | 3 |
| PGSI3 - Thinking about the past 12 months, how often have you gone back another day to try to win back the money you lost? (3) | 0 | 1 | 2 | 3 |
| PGSI4 - Thinking about the past 12 months, how often have you borrowed money or sold anything to get money to gamble? (4) | 0 | 1 | 2 | 3 |
| PGSI5 - Thinking about the past 12 months, how often have you felt that you might have a problem with gambling? (5) | 0 | 1 | 2 | 3 |
| PGSI6 - Thinking about the past 12 months, how often have people criticized your betting or told you that you had a gambling problem, regardless of whether or not you thought it was true? (6) | 0 | 1 | 2 | 3 |
| PGSI7 - Thinking about the past 12 months, how often have you felt guilty about the way you gamble, or what happens when you gamble? (7) | 0 | 1 | 2 | 3 |

CPGI_SCORE = PGSI1 + PGSI2 + PGSI3 + PGSI4 + PGSI5 + PGSI6 + PGSI7 + PGSI8 + PGSI9

***Interview Schedule – affected others***

1. **Affected by someone else’s gambling**

- Have you been negatively affected by someone else’s gambling?
- Who were they?
- What was the main type of gambling they were doing when you were negatively affected?
- In what way/s has this person’s gambling affected you?
- Did you seek help? If so, from where/who? If not, what were the reasons?

**2. Who is appropriate to approach people about gambling (not necessarily problems)?**

I’m going to ask you a few more questions….

- Who do you think might be appropriate to approach people about their gambling? This might include offering help or assistance with help-seeking for gambling problems (other than venue staff/betting company).
- Separately ask about:
- partner, other family
- friends,
- colleagues
- Services (e.g. GPs, health services, counsellors, welfare, financial counsellors)
- Anyone else?
- Are any of these inappropriate?

**3. Who would be the best to approach - someone, how, where, when?**

One of the things we want to find out is how to best approach someone about their gambling, so things might not get so bad. We know some people can find it uncomfortable to approach someone to help.

- Who do you think would be best placed to help people ‘so things don’t get so bad’?
- What do you think would be best to say?
- In a venue?
- While gambling?
- How bad would things need to be for you to say something?

**4. Other strategies and intervention*s***

- Aside from accessing services, what else might help people so things don’t get so bad (venues, partners, family, friends, etc.)?
- What do you think might encourage people to get help?
- Do you think there is a way that would make you/other people feel more comfortable talking about gambling?

***Ask at the end of the interview -*** What do you think governments could do to minimise harms from gambling?
